# The two facets of gp130 signalling in liver tumorigenesis

**DOI:** 10.1007/s00281-021-00861-0

**Published:** 2021-05-28

**Authors:** Dirk Schmidt-Arras, Eithan Galun, Stefan Rose-John

**Affiliations:** 1 Institute of Biochemistry, Christian-Albrechts-University Kiel, Kiel, Germany; 2 Goldyne Savad Institute of Gene Therapy, Hadassah Medical Centre, Hebrew University Jerusalem, Jerusalem, Israel

**Keywords:** IL-6, gp130, STAT3, Hepatocellular carcinoma (HCC), Cholangiocarcinoma (CCA), Tumour micro-environment, Anti-tumour immunity, Inflammation

## Abstract

The liver is a vital organ with multiple functions and a large regenerative capacity. Tumours of the liver are the second most frequently cause of cancer-related death and develop in chronically inflamed livers. IL-6-type cytokines are mediators of inflammation and almost all members signal via the receptor subunit gp130 and the downstream signalling molecule STAT3. We here summarize current knowledge on how gp130 signalling and STAT3 in tumour cells and cells of the tumour micro-environment drives hepatic tumorigenesis. We furthermore discuss very recent findings describing also anti-tumorigenic roles of gp130/STAT3 and important considerations for therapeutic interventions.

## Introduction

### The family of IL-6-type cytokines

The cytokine Interleukin-6 (IL-6) was originally cloned as a B-cell stimulating factor [1] but was subsequently shown to be identical with hepatocyte stimulating factor [2], indicating that the cytokine may have very different activities within the human body. Today, we know that IL-6 is not only important for the activation of the immune system and the orchestration of innate and acquired immune response [3, 4] but also plays a role in the maintenance of the central nervous system [5] and in the regulation of metabolism [6, 7].

**Fig. 1:**
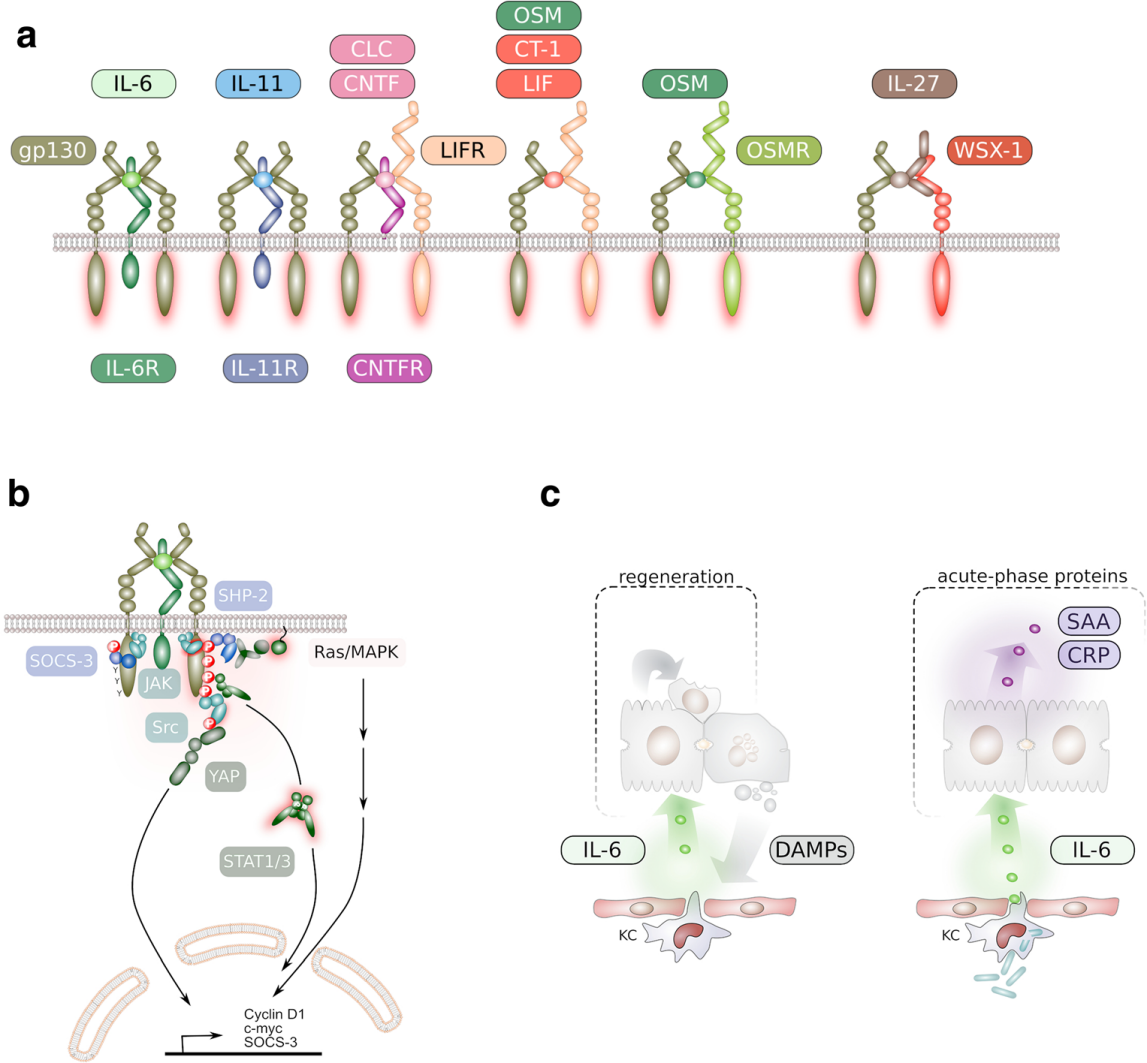
Physiological role of IL-6/gp130 in the liver. **a** Receptor complex formation of IL-6 family cytokines. IL, interleukin; CLC, cardiotrophin-like cytokine; CNTF, ciliary neurotrophic factor; CT-1, cardiotrophin-1; OSM, oncostatin M; LIF, leukaemia inhibitory factor; WSX-1, IL-27 receptor subunit alpha. **b** Major signal transduction pathways initiated by the IL-6/IL-6R/gp130 receptor complex. Receptor complex formation is followed by activation Janus kinases (JAK) that phosphorylate tyrosine residues on gp130 and on recruited downstream molecules. Recruitment of SOCS3 blocks JAK activity and therefore downstream signalling. MAPK, mitogen activated protein kinase; STAT, signal transducer and activator of transcription; YAP, yes-associated protein; SHP-2, Src homology region 2 domain-containing phosphatase-2; SOCS3, suppressor of cytokine signalling 3. **c** Major physiological functions of IL-6 in the liver. During regeneration, IL-6 induces proliferation and hypertrophy of hepatocytes (left side). IL-6 is the major inducer of acute-phase proteins in the liver upon infection (right side)

Biochemically, IL-6 is a four-helical protein with a typical up-up-down-down topology, which is shared by many cytokines [8, 9]. On target cells, IL-6 binds to the IL-6 receptor (IL-6R)α, which belongs to the class of hematopoietic receptors [8]. The complex of IL-6 and IL-6R α associates with a second receptor protein, glycoprotein 130 kDa (gp130), which upon dimerisation initiates signal transduction within the cell (Fig. 1a) [10]. Interestingly, gp130 is also a signalling receptor of the cytokines IL-11, IL-27, IL-35, ciliary neurotrophic factor (CNTF), cardiotrophin-1 (CT-1), leukaemia inhibitory factor (LIF), oncostatin M (OSM), and cardiotrophin-like cytokine (CLC) (Fig. 1a) [9]. These cytokines form the family of IL-6-type cytokines [9]. Of these cytokines, LIF, OSM, and CNTF have been identified as additional hepatocyte stimulating factors responsible for the induction of the hepatic acute-phase protein induction [11]. Consequently, intracellular signal transduction pathways of all these cytokines are very similar although not identical [12].

### gp130 signal transduction

Dimerisation of gp130 by the IL-6 and IL-6R α complex leads to activation of the tyrosine kinase JAK1, which is constitutively bound to gp130. JAK1 phosphorylates the five tyrosine residues within the cytoplasmic portion of gp130. The membrane proximal tyrosine is the docking site for the phosphatase SHP2, which initiates the MAPK pathway and the PI3K pathway (Fig. 1b). The four membrane distal phosphotyrosine residues recruit STAT1 and STAT3, which upon phosphorylation homo- or heterodimerise and translocate to the nucleus where they act as transcription factors for STAT target genes (Fig. 1b). One of the earliest STAT target genes codes for the protein SOCS3, which is recruited to the membrane proximal tyrosine residue from where it inhibits JAK1 activity and prevents SHP2 binding (Fig. 1b). Thereby, SOCS3 provides negative feedback inhibition of gp130 activation [10]. In addition to the above mentioned signalling pathways, it was found that Src family tyrosine kinases are recruited to the cytoplasmic portion of gp130 and that this signal transduction is independent of receptor- and STAT3-phosphorylation. Src family kinases phosphorylate the transcriptional co-activator YAP (yes-associated protein) leading to activation of YAP target genes and tissue growth [13]. Interestingly, the JAK/STAT pathway and the YAP pathway are strongly activated in the regenerating liver [13, 14].

### The cellular landscape of IL-6 family cytokine sender and receiver in the liver

The liver consists of different cell types. Hepatocytes, the liver parenchymal cells represent the largest cellular mass of the liver and fulfil multiple functions, including a central function in body metabolism, detoxification, and the synthesis of bile and plasma proteins. Biliary epithelial cells (BECs) are lining the intra- and extrahepatic bile ducts. Both epithelial lines can be the origin of hepatic tumours. The liver also harbours different inflammatory cells of the adaptive and the innate immune system, among them the Kupffer cells (KCs), that are liver-resident macrophages derived from the foetal yolk sac. Hepatic stellate cells (HSCs) are found in the perisinusoidal space and serve as lipid and vitamin A storage. Upon liver damage, HSCs differentiate into collagen-secreting myofibroblasts [15].

Under physiological conditions, expression of IL-6 family cytokines is barely detectable in the liver. However, upon infection, challenge with microbial antigens or tissue damage, levels of IL-6 and IL-11, and OSM can increase tremendously. Under these conditions, myeloid cells, in particular, KCs are the major source of IL-6 and OSM. However, it was also demonstrated that non-parenchymal cells recruited via IL-17 are important sources of IL-6 during liver regeneration [16] and hepatic fibrosis [17]. During chronic liver disease also senescent hepatocytes and BECs secrete IL-6 (see also below), while OSM is also secreted by hepatic progenitor cells [18]. IL-11 is secreted by activated HSCs [19] [20] and lipid-loaded hepatocytes [20] in the course of non-alcoholic fatty liver disease (NAFLD).

The signal-transducing subunit gp130 of IL-6 family receptor complexes is ubiquitously expressed in the body. Expression of gp130 in hepatocytes seems to be downregulated by bile acids which may contribute to hepatocyte death during cholestasis [21]. While gp130 is ubiquitously expressed in the liver, response to a particular cytokine family member is limited by the expression of its cognate α receptor.

IL-6 needs to bind to the IL-6R α in order to induce dimerisation of gp130 and initiation of intracellular signalling [9]. The membrane-bound IL-6R α is subject to limited proteolysis by proteases such as ADAM10 and ADAM17 [22, 23], and in this way, generated soluble IL-6R α still binds IL-6 and can elicit IL-6 signals on cells, which do not express IL-6R α [9]. This mode of signalling has been named IL-6 trans-signalling [24]. The IL-6 trans-signalling pathway not only vastly enlarges the spectrum of IL-6 target cells but also increases the signalling strength and prolongs IL-6 signals on cells, which do express the IL-6R α [25] due to the typically higher expression of gp130 as compared to IL-6R α [9].

In the liver, all cell types are able to respond to IL-6, and expression of IL-6R α was detected on hepatocytes, BECs [26], and HSCs [27]. Using a novel mouse model of cell-autonomous gp130 activation, we recently showed that hepatocytes but not BECs or HSCs react most prominently to gp130 activation [28]. However, this does not exclude that, under pathological conditions, IL-6-type cytokines regulate biological behaviour of BECs or HSCs.

### Physiological role of IL-6 family cytokines in the liver

IL-6 regulates multiple functions in the liver, including infection defence, metabolism, and regeneration. In the acute phase of an infection, plasma levels of inflammatory cytokines such as TNFα, IL-1β, and IL-6 sharply increase, followed by enhanced secretion of proteins belonging to the family of acute-phase proteins (Fig. 1c) [29]. These proteins are able to prevent systemic spreading of an infection by pathogen opsonisation, enhancing blood coagulation and complement activation and the initiation of adaptive immunity. The necessity of IL-6/gp130 signalling for the induction of acute-phase proteins was initially demonstrated in mice deficient for IL-6 [30]. By using mice either deficient for gp130 [31–33] or with cell-autonomous gp130 activation [28], it was shown that gp130 activation in hepatocytes is sufficient to trigger acute-phase proteins secretion. There is experimental evidence that production of acute-phase proteins is even enhanced by IL-6 trans-signalling [28, 34, 35]. Recently, inactivating mutations in *IL6ST*, encoding gp130 [36] and inactivating mutations in *IL6RA* [37] were observed and demonstrated that, also in humans, IL-6/gp130 signalling is essential for the secretion of acute-phase proteins. Through the induction of hepcidin in hepatocytes, IL-6/gp130 signalling impairs ferroportin-mediated iron release from intestinal epithelial cells to further dampen bacterial infections [38, 39]. Furthermore, gp130 signalling in hepatocytes induces the mobilisation and recruitment of neutrophils via the secretion of the neutrophil attractant CXCL1 in mice or the functional orthologue IL-8 in humans [28, 33, 40].

The liver has a unique capacity to regenerate and IL-6 was identified as a major driver of liver regeneration. Shortly after hepatectomy, liver vein levels of TNFα increase, followed by a strong induction of IL-6 [41]. Consistently, IL-6-deficient mice display impaired liver regeneration [42]. IL-6 promotes liver regeneration by two means: prevention of hepatocyte cell death and stimulation of hepatocyte proliferation (Fig. 1c). While IL-6-deficient mice display a marked reduction in hepatocyte proliferation [42], administration of recombinant IL-6 acts as a direct hepatocyte mitogen [43]. This indicates that albeit other growth factors such as HGF contribute to liver regeneration, IL-6 is a key regulator of liver regeneration. IL-6/gp130 signalling was shown to prevent hepatocyte apoptosis upon DNA damage through stabilisation of Mcl-1 and the prevention of p53 stabilisation [44, 45]. Activation of the PI3K/AKT pathway contributes to the anti-apoptotic effect of IL-6/gp130 signalling in hepatocytes [46, 47].

Albeit hepatocytes express IL-6R α and therefore respond to IL-6 classic signalling, hepatocyte proliferation, and hence, liver regeneration is enhanced by IL-6 trans-signalling. This observation can be explained by the fact that hepatocytes express far more gp130 than IL-6R α. Consequently, in the presence of IL-6R α and soluble IL-6R α (sIL-6R), a larger fraction of gp130 molecules is stimulated than by IL-6 alone [46, 48–50].

While earlier reports demonstrated that recombinant human IL-11 protects from hepatocyte damage induced by oxidative stress, drugs, or ischemia/reperfusion [51–54], more recent reports show that IL-11 rather promotes liver damage via ROS production [20], activates HSCs [19, 20], and hence, promotes liver fibrosis in the setting of chronic liver disease. Similarly, OSM was shown to prevent hepatocyte damage by oxidative stress [55–57] and to promote hepatic fibrosis by upregulation of TGFβ and PDGF in hepatic macrophages [58], and by stimulating myofibroblast migration [59].

## Pro-tumorigenic roles of IL-6/gp130

### Regulation of IL-6 secretion during tumorigenesis

Hepatic tumours, in particular HCC, are classical examples of inflammation-driven cancers [60], and composition of the tumour-associated immune compartment is key to carcinogenesis and metastasis [61]. Tumour-associated myeloid cells, in particular KCs, were identified as major source for IL-6 [62, 63]. Expression and secretion of IL-6 in KCs are suppressed by activated oestrogen receptor (ER) α which explains at least in part the gender disparity in HCC formation in humans [62].

A common requirement for the secretion of IL-6 from tumour-associated macrophages (TAM) of the intestine and the liver is an autocrine EGFR activation loop [64, 65]. Interestingly, EGFR overexpression [66] and upregulation of EGFR ligands such as transforming growth factor (TGF) α [67] and epiregulin (EREG) [68] were reported in human and murine HCC.

**Fig. 2:**
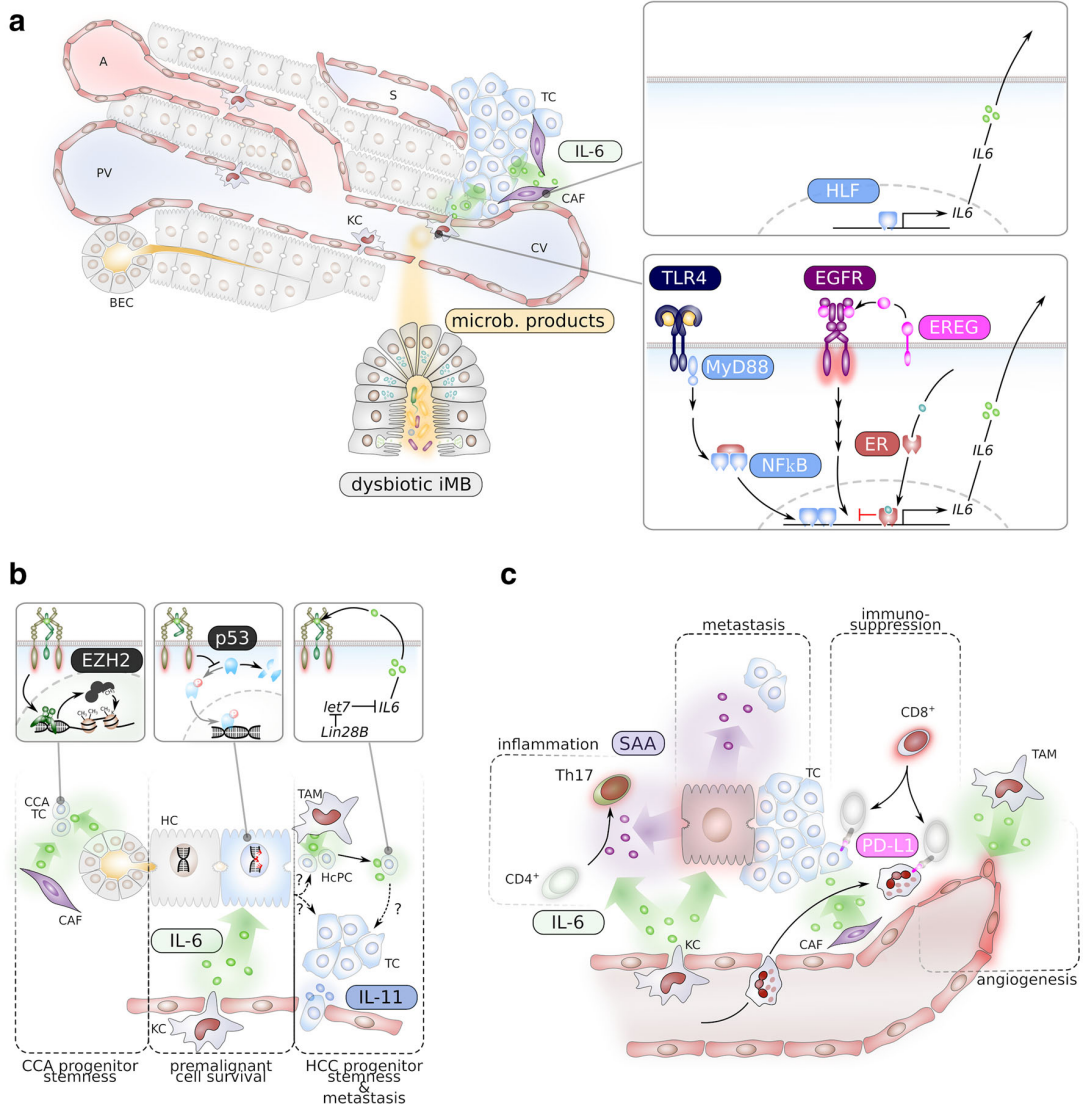
Pro-tumorigenic role of IL-6/gp130 signalling in the liver. **a** Mechanisms of IL-6 upregulation in cancer-associated fibroblasts (CAFs, upper right panel) and myeloid cells (lower right panel). **a** Hepatic artery; BEC, biliary epithelial cells; CV, central vein; HLF, hepatic leukaemia factor; EGFR, epidermal growth factor receptor; EREG, epiregulin; ER, oestrogen receptor; HC, hepatocyte; iMB, intestinal microbiota; MyD88, KC, Kupffer cell; NFkB, nuclear factor k B; PV, periportal vein; S, sinusoid; TC, tumour cell; TLR4, toll-like receptor 4. **b** Direct cancerogenic effects of IL-6 and IL-11 on tumour cells. CCA, cholangiocarcinoma; EZH2, enhancer of zeste homolog 2; HcPC, HCC progenitor cells; let7, lethal 7; Lin28B, Lin28 homolog B; TAM, tumour-associated macrophages. **c** IL-6 promotes tumorigenesis and metastasis via the induction of an immunosuppressive tumour micro-environment. PD-L1, programmed cell death 1 ligand 1; SAA, serum amyloid A

Obesity is linked to an increased risk of tumour development and was shown to promote HCC formation via enhanced TNF α and IL-6 secretion [69]. Alterations in the intestinal microbiota composition called “dysbiosis” are common to obesity, age-dependent inflammation [70], and chronic liver disease [71]. Venous blood that drains from the intestine first passes the liver and KCs serve as gatekeepers that protect against intestinal-derived pathogens. Microbiota-associated molecular patterns (MAMPs) are sensed by toll-like receptors (TLR) on KCs leading to recruitment of the adaptor molecule MyD88 and/or TIR domain-containing adapter molecule 1 (TICAM-1/TRIF) and activation of downstream signalling (Fig. 2a). It was shown that, during HCC formation, intestinal dysbiosis enhanced EREG secretion in a TLR4-dependent manner [68]. It is therefore not surprising that KC-mediated IL-6 secretion during hepatic carcinogenesis is hampered in mice deficient for MyD88 or toll-like receptor (TLR) 4 [62, 72]. In this context, the serine-threonine protein kinase (STK) 4 counteracts TLR signalling and concomitant IL-6 secretion through phosphorylation of the TLR downstream signalling molecular IRAK1 [73] and is therefore considered as tumour suppressor for HCC [74].

B-Cells are present in HCC [61] and were shown to undergo immunoglobulin class switch recombination [75]. IgG secreted by these plasma cells binds to Fcγ receptor on TAMs thereby enhancing IL-6 secretion [75]. Similarly, BEC autoreactive antibodies in patients with primary sclerosing cholangitis, a chronic liver disease that predisposes to cholangiocarcinoma formation, bind to and induce secretion of IL-6 from BECs [76].

Cancer-associated fibroblasts (CAFs) are key components of the tumour micro-environment [77]. Also in the liver, HCC often develops in cirrhotic liver that is promoted by activated HSCs [15, 78]. During liver fibrosis, HSCs are a cellular source of IL-6, and *IL6* transcription is enhanced via the hepatic leukaemia factor (HLF) transcription factor (Fig. 2a) [27]. CAFs isolated from prostate cancer [79] and intrahepatic cholangiocarcinoma were also shown to highly secrete IL-6 [80].

Senescent cells secrete diverse molecules, including cytokines such as IL-6, which is called the senescence-associated secretory phenotype (SASP). During chronic liver disease, hepatocytes can become senescent. Both senescent hepatocytes [81, 82] and cholangiocytes [83] were shown to secrete IL-6. Consequently, surveillance of senescent hepatocytes is the key to the prevention of HCC formation [84]. But also non-senescent hepatocytes were shown to upregulate *IL6* expression via co-binding of nuclear factor (NF)-κB and the polycomb repressor complex (PRC) 2 member enhancer of zeste homolog (EZH) 2 [85].

Beside paracrine activation, hepatic tumour cells can encounter cell-autonomous activation of gp130. Inflammatory hepatocellular adenoma (IHCA) is a benign form of hepatic tumours and is characterised by the accumulation of inflammatory cells, including B-cells [86, 87]. In most of IHCA cases, constitutive activation of the gp130/STAT3 pathway has been found, including activating deletion mutations *IL6ST*, encoding gp130 [87], and activating point mutations in *STAT3* [88]. However, while activating mutations of *IL6ST* are found in 60% of IHCA cases, they are detectable only in a small fraction of HCC tumours. Nevertheless, persistent gp130 activation was found in murine HCC progenitor cells (HcPCs), tumour cells that express typical markers of hepatic progenitor cells. Persistent activation of gp130 in these cells was mediated via autocrine IL-6 secretion which was upregulated by LIN28 [89]. Similarly, an autocrine IL-11 loop is established by a TGFβ-induced long non-coding (lnc) RNA-ATB in metastasing HCC (Fig. 2b) [90].

### Effect of IL-6 on (pre-)malignant hepatic cells

Serum levels of IL-6 are high in chronic liver disease predisposing to hepatocarcinogenesis, suggesting that IL-6/gp130 signalling is a major driver of hepatocarcinogenesis. And indeed, not only IL-6-deficient [62] mice but also mice with hepatocyte-specific gp130-deficiency [91] display strongly impaired tumour formation not only in a murine DNA damage-driven HCC model but also in an obesity-driven liver tumour model. Furthermore, hepatocyte carcinogenesis was shown to be accelerated via enhanced genomic instability [92, 93]. While impaired DNA damage response during chronic inflammation is mainly mediated by TNF α [70, 94], survival and proliferation of genomic unstable hepatocytes are driven by gp130 trans-signalling by preventing p53-induced hepatocyte apoptosis (Fig. 2b) [45, 93, 95]. Not only IL-6 but also IL-11 seems to contribute to gp130-driven carcinogenesis as it was demonstrated that recurrence of experimental HCC upon hepatectomy was impaired in *IL11ra*-deficient mice [96].

Albeit the cellular origin of HCC is still under debate, the occurrence of cells with a liver stem/progenitor cell phenotype was reported in human and experimental HCC [63, 89, 97] that were able to reconstitute hepatic tumours in transplantation experiments [89]. These cells were termed HCC progenitor cells (HcPCs). HcPCs seem to depend on inflammatory signalling, and ectopic lymphoid structures in the liver were shown to promote survival and outgrowth of HcPCs [97]. During an early stage of hepatocarcinogenesis, HcPCs depend on paracrine IL-6 derived from KCs or TAMs [63, 89], while at a later stage of carcinogenesis, HcPCs develop an autocrine IL-6 loop through Lin28B-mediated suppression of the inhibitory miRNA *let7* (Fig. 2b) [89]. Furthermore, in metastatic HCC, establishment of an autocrine IL-11 loop promotes metastatic colonisation (Fig. 2b) [90].

Similar to carcinogenic hepatocytes, proliferation and stemness of intrahepatic cholangiocarcinoma (CCA) cells are enhanced by IL-6/gp130 signalling through the upregulation of EZH2 that mediates histone H3 methylation (Fig. 2b) [80]. This correlates with enhanced expression of gp130 and IL-6R α in CCA cells as compared to BECs [98]. The observation that proliferation of human CCA cell lines is reduced in the presence of a neutralising anti-IL-6 antibody [98] suggests that CCA cells also can adopt autocrine IL-6/gp130 signalling.

The fact that constitutive activation of the gp130/STAT3 pathway is found in inflammatory liver adenoma and hepatic tumour progenitor cells suggests that constitutive activation of gp130 in hepatocytes or liver progenitor cells is sufficient to drive liver tumorigenesis. In order to address this hypothesis, we previously generated an artificial constitutive active gp130 variant by replacing the extracellular domain of gp130 with the c-Jun leucine zipper [99], which we termed “Lgp130”. We generated mice with a Cre-inducible expression cassette in the ROSA26 locus. When Lgp130 was expressed in B-cells, it was sufficient to drive B-cell malignancies [100]. However, when Lgp130 was expressed in hepatocytes, we did not observe tumour formation in aged mice, despite persistent gp130/STAT3 activation [28]. These data suggest that gp130/STAT3 signalling alone does not confer malignant transformation. However, constitutive gp130 signalling was able to promote oncogenic transformation in human foetal hepatocytes when combined with DNA double strand breaks [95].

### Effect of IL-6 on tumour micro-environment

The tumour micro-environment of hepatic tumours is composed of different inflammatory cells, and there is growing interest in the application of immunotherapeutic in hepatic cancers [61]. For the detailed inflammatory composition of HCC tumour micro-environment, the reader is referred to recent excellent reviews [61, 101, 102]. There is increasing evidence that IL-6 family cytokines are involved in shaping the inflammatory tumour micro-environment in hepatic cancers. Different CD4^+^ T helper (Th) subpopulations, including Th17, were recently identified in tumoural and peritumoural tissue and described to exert a pro-tumorigenic function [103–106]. IL-6 was previously shown to trigger Th17 differentiation in combination with TGFβ by the upregulation of IL-21, and the establishment of an autocrine IL-21 loop resulting in stable STAT3 activation that in combination with RAR-related orphan receptor (ROR) γt is necessary for the expression of *IL17* [107]. Very recently, it was demonstrated that pathogenic pro-inflammatory Th17 in the intestine are induced by STAT3-activating cytokines in combination with serum amyloid A (SAA) proteins that are secreted by adjacent intestinal epithelial cells [108]. Given the fact that gp130/STAT3 activation in hepatocytes is sufficient to induce SAA1 and 2-secretion [28], it is likely that, also in HCC, the appearance of pro-tumorigenic Th17 cells is orchestrated by IL-6 (Fig. 2c).

Expression of inhibitory molecules including programmed cell death protein (PD)-1 and T cell immunoglobulin and mucin domain (TIM) 3 is increased on CD4^+^ and CD8^+^ T-cells in HCC tissue [61]. Inhibitory molecules on T cells guard against autoreactivity but are also a sign of T cell exhaustion, a state of lymphocyte dysfunction. Tumour cells use this mechanism to evade surveillance through the adaptive immune system. IL-6 was shown to promote expression and stability of T cell inhibitory molecules. In HCC cell lines, IL-6 increased the surface localisation of PD-L1 (Fig. 2c), the ligand for the inhibitor molecule PD-1. gp130/JAK activation induced PD-L1 phosphorylation, and in turn, altered glycosylation that resulted in an enhanced stability of PD-L1 on the cell surface [109]. Furthermore, CAFs isolated from HCC were shown to recruit and activate neutrophils [110] via secretion of IL-6 and induction of STAT3 activity in neutrophils (Fig. 2c). These activated neutrophils [110], also myeloid-derived suppressor cells [85], dampened an anti-tumour T-cell response through the upregulation of PD-L1 (Fig. 2c). Similarly, IL-6 derived from glioblastoma cells induced PD-L1 in tumour-associated myeloid cells [111]. It is therefore not surprising that combination of anti-PD-1 antibodies with anti-IL-6 antibodies impairs the immunosuppressive tumour micro-environment and is a promising strategy also for the therapy of HCC [110, 112, 113].

Interestingly, it was recently shown in a murine model of primary sclerosing cholangitis that IL-17 from Th17 cells promotes the expression of PD-L1 on BECs [114] thereby not only dampening auto-inflammation on one side but also potentially preventing proper anti-tumour response in cholangiocarcinoma.

KCs were suggested to promote survival of liver sinusoidal cells in an IL-6/gp130-dependent manner [115]. Accordingly, it was shown that tumour vascularisation in murine HCC models is enhanced by IL-6 trans-signalling [45, 116] and thereby further promoting hepatic tumourigenesis (Fig. 2c).

IL-6 signalling also plays an essential role for the preparation of a hepatic metastatic niche. On one hand, IL-6 induced PD-L1 expression on colorectal cancer cells thereby blunting anti-tumour effector function of CD8^+^ T cells [117]. On the other hand, gp130/STAT3-dependent secretion of SAA proteins by hepatocytes promoted metastatic colonisation of pancreatic cancer cells in the liver [118].

### The impact of other IL-6 family cytokines on hepatocarcinogenesis

While there is clear evidence that IL-6 contributes to hepatic tumour formation, the role of other IL-6 family cytokines is less clear. However, there is evidence that OSM and CLC contribute to hepatic tumorigenesis, while LIF and IL-27 rather seem to play a tumour suppressive role.

The OSMR is expressed on HcPCs and proliferation and hepatocytic differentiation of these cells is promoted by OSM [119]. Neutrophils that accumulate in hepatic tumour tissue produce OSM upon paracrine stimulation with TNFα secreted by TAMs [120]. As a consequence, OSM is hypothesized to promote hepatocarcinogenesis and intrahepatic metastasis. CLC, secreted by CAFs, was recently identified to accelerate hepatocellular carcinogenesis [121] and engagement of CNTFR induces MAPK activation in HCC cell lines in vitro [122].

Expression of LIFR is lost during malignant progression of hepatic tumours [123], suggesting that LIF plays a tumour suppressive role. However, little is known on the underlying mechanisms.

Expression of IL-27 is upregulated in HCC patients [124]. In HCC cell lines, IL-27 induces robust STAT1 rather than STAT3 phosphorylation and a STAT1-dependent expression profile [125]. As a consequence, IL-27 induced expression of MHC I, suggesting more effective antigen presentation, but also, expression of PD-L1 was elevated [126]. However, the effect of IL-27 on growth of hepatic tumours in vivo is far from being understood, as IL-27 did not prevent the orthotopic growth of an HCC cell line in mice [127].

## Anti-tumorigenic roles of IL-6/gp130/STAT3

The fact that IL-6 is a pleiotropic cytokine with a unique ligand–receptors interaction and natural “built-in” shed and intracellular inhibitors make it not unexpected that its effect on tumorigenesis is context-dependent and not “monochromatic”. Although most investigations show the pro-tumorigenic effect of IL-6, it also encounters several properties that directly or indirectly execute its anti-tumorigenic properties.

Numerous mechanisms and associations were reported between increased IL-6 expression and signalling and levels and suppression of tumorigenesis. These include the following: 1. the role of IL-6 in liver fibrosis, 2. the role of IL-6 in senescence, and 3. the tumour suppressive effects of STAT3.

### Prevention of hepatic fibrosis

**Fig. 3:**
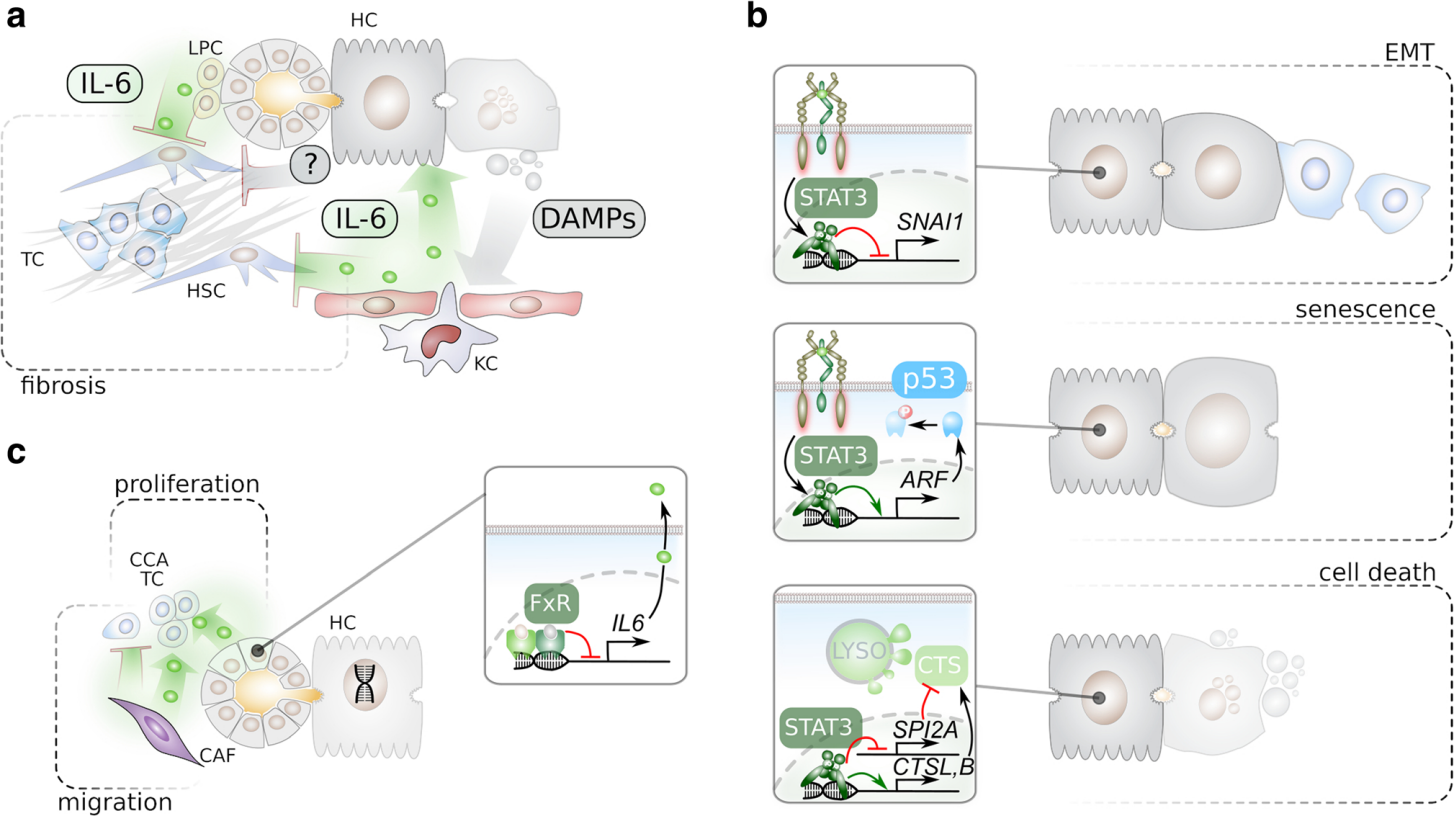
Anti-tumorigenic effects of IL-6/gp130 signalling. **a** IL-6, secreted by KCs or LPCs, prevents hepatic fibrosis and thereby reduces the risk of HCC development through enhancing hepatocyte repair/proliferation and inhibition of HSCs. DAMPs, death-associated molecular patterns; HSC, hepatic stellate cell; LPC, liver progenitor cell. **b** IL-6 can have direct anti-tumorigenic effects by (I) inhibiting EMT, (II) induction of p53-mediated senescence, or (III) the induction of cathepsin-mediated cell death. ARF, alternative reading frame; CTSL, B, cathepsin (CTS) L, B; EMT, epithelial-to-mesenchymal transition; LYSO, lysosome; SNAI1, snail homolog 1; SPI2A, serine protease inhibitor 2A. **c** While IL-6 enhances survival and proliferation of cholangiocarcinoma cells, it prevents migration and invasion. Activation of FxR in cholangiocytes prevents the secretion of IL-6. CAF, cancer-associated fibroblast; CCA, cholangiocarcinoma; FxR, farnesoid X receptor; HC, hepatocyte; TC, tumour cell

Fibrosis is a complexed condition involving different cytokines, including IL-6 [128]. Liver fibrosis is perceived as a contributing factor to the development of liver injury, and vice versa, liver injury, which is usually the initiating event, causes the development of liver fibrosis [129]. Furthermore, fibrosis is a significant factor for liver disease outcome and a risk for the development of hepatocellular carcinoma (HCC) [78]. It was already shown 20 years ago that IL6 deficiency causes enhanced liver fibrosis upon the development of liver injury [130]. IL-6 KO mice are shown to be more susceptible to liver steatosis and injury under a high-fat diet [131, 132]. In a CCl_4_ model of liver fibrosis, the attenuation of fibrosis by sorafenib correlated with increased STAT3 phosphorylation in hepatocytes which was dependent on KC-derived IL-6 [133]. In addition, it was shown that, upon deletion of STAT3 in hepatocytes, there is an exacerbation of liver fibrosis during cholestasis. Unidentified factors released from hepatocytes, dependent on STAT3, play a protective role in liver fibrogenesis through an inhibitory effect on activated HSCs (Fig. 3a) [134]. The mechanism of how IL-6 prevents and reverses hepatic fibrosis is still under investigations. One proposed mechanism is that bipotential murine oval liver cells, thought to be hepatic progenitors, secret IL-6 which could induce the apoptosis of HSCs [135]. In alcoholic liver disease in humans, it was also suggested that IL-6 has an anti-fibrotic effect through the STAT3 signalling pathway [136]. An additional potential mechanism is through the inhibition of inflammation in specific cases, as was reported in the lipopolysaccharide/d-galactosamine (LPS/d-Gal)-induced acute liver injury in rodent model, in which IL-6 has an anti-injury property [137]. Alcoholic liver disease is associated with HCC [138]. The protective role of IL-6 was also shown in an ethanol-induced oxidative stress model in which hepatocytes via induction of metallothionein protein expression dependent on IL-6 were protected against ethanol injury also by other mechanisms [139, 140].

### Direct anti-tumour effects

The pleiotropic nature of IL-6 mediates many cellular phenotypes, which are context-dependent. These are involved in metabolism, differentiation, and survival. Heme oxygenase-1 (HO-1) has a number of anti-injury properties mediated by catabolic by-products such as biliverdin, which suggests that HO-1 is a tissue protector. A recent report shows that HO-1 is a tumour suppressor gene, which is induced by IL-6 [141].

STAT3, although perceived as a traditional target for treating cancer, until today, this is not translated into clinical usage [142]. This is also true for the use of STAT3 inhibitors for the treatment of HCC. None of the STAT3 inhibitors passed phase III clinical studies for HCC. Lysosomes are recognized today as pivotal in many cellular processes. Cellular transformation is associated with lysosomal modifications, potentially also promoting tumorigenesis [143]. STAT3 mediates lysosomal-mediated programmed cell death in mammary epithelial cells, by formation of large vacuoles containing triglyceride, inducing leakage of cathepsins which culminates in cell death [144]. Altogether, this teaches us that STAT3 phosphorylation downstream to IL-6 signalling could suppress breast cancer development and progression.

The role of STAT3 is also dichotomic. In the liver, STAT3 is activated in cholangiocytes enhancing cholangiocytic cancer stem cell for proliferation downstream to the signalling of CD24 and NANOG [145]. Loss of STAT3 in lung and pancreatic cancers was associated with mesenchymal transition of epithelial cells and an aggressive tumour phenotype. Whereas, STAT3 activation conferred a differentiated cells epithelial phenotype and reversed the cancerous phenotype [146]. STAT3 was also shown to encounter tumour suppressive effects in other types of tumours including papillary thyroid carcinoma [147], glioblastoma [148], in colon carcinoma STAT3 suppresses the development of Apc^Min^ cancer possibly through the downregulation of Snail-1, suppressing an epithelial-mesenchymal transition of colorectal cancer cells [149, 150]. A similar observation was reported in KRAS mutation induced lung adenocarcinoma, in which disruption of STAT3 induced tumorigenesis [151]. Furthermore, in smokers with KRAS mutation, lung adenocarcinoma STAT3 correlated with poor survival and advanced malignancy. The experience and disappointment with STAT3 inhibitors were also apparent for prostate cancer. Prostate cancer is the most frequent cancer in males, and the phosphatase and tensin homologue (PTEN) gene is the most frequently mutated gene in this malignancy. Mice with a conditional mutation of PTEN in the prostate epithelium are a commonly used mouse model for prostate cancer. Unexpectedly, genetic inactivation of STAT3 or IL-6 in prostate-specific PTEN knock-out mice led to accelerated tumour progression and metastasis [152]. This result helped to explain the result from clinical trials, in which patients with advanced prostate cancer were treated with a neutralizing IL-6 antibody without any significant survival advantage [153]. In the prostate-specific PTEN knockout mouse model, it was shown that the loss of IL-6/STAT3 signalling bypassed cellular senescence by disrupting the ARF-p53 axis indicating that alternative reading frame protein (ARF) was a novel STAT3 target gene [152]. In line with the animal studies, it was shown in prostate cancer patients that loss of STAT3 and ARF correlated with increased risk of tumour recurrence. These results yield a molecular explanation how the IL-6/STAT3 axis, which in many tumours has an oncogenic potential, can also act in the maintenance of senescence and thereby act as a tumour suppressor (Fig. 3b) [152].

In a Myc-dependent breast cancer mouse model, STAT3 deficiency was associated with enhanced epithelial-to-mesenchymal transition and metastasis, indicating a potential anti-metastatic property of STAT3 [154]. In summary, although STAT3 is perceived a pro-tumorigenic mediator of signalling upon its phosphorylation, growing number of reports teach to the fact that the role of STAT3 in tumorigenesis is more context-dependent.

### Effect of IL-6/gp130 signalling on CCA

Intrahepatic cholangiocarcinoma is a very aggressive cancer and the second most common among liver cancers. Recent publications report quite controversial findings on the role of IL-6 in cholangiocarcinoma. IL-6 is proposed to be secreted from CAFs of this tumour, inducing epigenetic changes in cholangiocytes and thereby enforcing a malignant transformation driving the initiation of intrahepatic cholangiocarcinoma [80]. However, a recent report observed a negative correlation between IL-6 levels and intrahepatic cholangiocarcinoma [155]. In addition, farnesoid X receptor (FXR), which is downregulated in intrahepatic-cholangiocarcinoma cell lines and human samples, has a negative correlation with aggressiveness and poor prognosis of patients with intrahepatic-cholangiocarcinoma. FXR expression was negatively correlated with IL-6 in intrahepatic cholangiocarcinoma tissues. FXR inhibited intrahepatic cholangiocarcinoma aggressiveness through the suppression of IL-6 [156]. However, it was shown that inhibition of IL-6 trans-signalling by the administration of recombinant sgp130Fc reduced cholangiocarcinoma cell line viability and induced apoptosis, whereas both migration and proliferation increased [157]. In one type of cholangiocarcinoma, carcinoma of the gallbladder (GBC), IL-6R α (gp80), was downregulated and correlated positively with an improvement of overall survival. Overall, these complex observations of the role of IL-6 in human cholangiocarcinoma, showing both pro-tumorigenic and anti-tumorigenic properties, are “reproduced” in other types of cancers as well. These complexed observations render a simple therapeutic approach. This complexity imposes a case-by-case investigation and understanding prior to developing therapeutic approaches.

### Regulation of tumour cell senescence

Senescence is initiated following an external stress imposed on the tissue. In the liver, this could be inflammation, infection, or metabolic strain. Senescent cells arrest in the cell cycle, encounter morphological changes, and produce a specific and complex secretome, the senescence-associated secretory phenotype (SASP) [158]. The development of DNA damage leads to cell cycle arrest through the activation of p53, and the induction of p21^CIP1^ and p16^INK4a^ inhibits cyclin-dependent kinases CDK4, CDK6, and CDK2 in some cases. IL-6 is a major component of the SASP response although it is now known that SASP could harbour hundreds of protein and non-protein substances with inflammatory and immunological properties [159]. Induction of senescence in cancer opens an opportunity to treat the malignancy with senolytic agents that selectively induce cell death in senescent cells [160]. This was recently shown to be effective in liver cancer [161]. Although the role of IL-6 in senescence-induced anti-tumour effects was reported in non-HCC [162], the role of IL6 in senescence-mediated anti-tumour effects in different types of liver cancers is still under investigation and seems to be dependent on the tumour type (E.G., personal communication). However, in some pathological conditions upon stress, senescence develops, as in alcoholic liver disease. It was recently shown that M2 macrophages trigger hepatocyte senescence and enhance alcohol-induced hepatocyte senescence, as indicated by increased β-galactosidase activity, elevated CDKN1A mRNA expression, and induction of nuclear p21. This group identified IL-6 as the mediator of M2-induced hepatocyte senescence. Senescent hepatocytes might display protective effect against alcoholic liver disease, a pre-malignant condition upon becoming chronic [163].

## Therapeutic considerations

In the intensive investigations on the role of the IL-6/STAT3 pathway, although unfolded many mechanistic understandings related to the development of liver cancer and other malignancies, no single drug was yet approved that is based on these mechanistic findings. However, specific targets and approaches interfering with the IL-6/STAT3 pathway are highlighted and are potentially important to indicate in this review. The potential contribution of senescence to the development of HCC has been investigated in an effort to identify new therapeutic targets against HCC. Senolytic agents were shown to have a beneficial effect on HCC [161] but, at the same time, warranted further investigations [164].

There are some recent developments in the applications of kinase inhibitors (sorafenib [165] and lenvatinib [166]) and immunotherapies for HCC [61, 167]. However, these encounter many side effects and escapes from treatments and are currently indicated for a more advanced disease. Due to the intensive cancer surveillance programs worldwide, many small tumours are detected in patients with cirrhosis at relatively early stages [168]. For these patients, regional approaches including partial/segmental hepatectomy (PHx), transcatheter arterial chemoembolisation (TACE), and radiofrequency ablations (RFA) gained ground as an important approach for treating HCC local/regional disease [169]. However, these approaches are also associated with high recurrence frequency. We have shown in the MDR2 KO mice model [170], which simulates inflammation-induced chronic liver injury and cancer, that there is an enhanced hepatocarcinogenesis following PHx [92]. This occurs with enhanced DNA damage response, increased genomic instability, escape of cell-cycle arrest, and senescence and causes tumour growth acceleration subsequent to PHx, causing HCC recurrence. In a recent investigation, to unfold the enhanced carcinogenic effect of PHx, it revealed that, under these inflammatory conditions, there is a striking increase in hepatocytes bearing micronuclei, a marker of genomic instability, which is suppressed by IL-6 blockade [93]. The vast majority of patients in the western world develop HCC on the background of cirrhosis, rendering PHx as a preferred therapeutic approach. However, PHx in cirrhotic patients is associated with high mortality. This leads to the development of alternatives. RFA is a potential therapeutic approach for small size tumours in cirrhotic livers [171]. However, RFA is associated with HCC recurrence [172]. Based on these observations, we have recently dissected the mechanism of this recurrence in in vitro and in vivo models, showing a panel of inflammatory mediators responsible for enhanced hepatocyte proliferation and HCC recurrence in mouse models exposed to RFA, including STAT3, IL-6, c-MET, COX-2, and heat shock proteins [173–182]. All these are currently undergoing further investigation to identify the preferred therapeutic approach in combination of RFA to suppress HCC recurrence.

## Perspective, future research

In the past decades, IL-6 has emerged as an important mediator of tissue inflammation and regeneration. It was therefore not surprising that IL-6 and STAT3 which act as the major transcription factor downstream of the IL-6 receptor complex were initially considered tumour promoters in many cancer types including the liver. Several cell types and mechanisms in the tumour micro-environment of the liver were identified to regulate the expression and secretion of IL-6.

However, more recent research has shed more light on the complexity of IL-6 signalling in cancer including liver. It turned out that IL-6 not only has tumour-promoting effects but acts also in tumour prevention. Therefore, future research has to unveil a more detailed picture on the kinetics and cellular context of IL-6 signalling in order to precisely distinguish between pro- and anti-tumorigenic effects of IL-6 signalling. This might include epigenetic mechanisms, the identification of co-dependencies, and a more detailed understanding of its role in anti-tumour immunity.

Consequently, we will be able to design novel therapeutics that are able to block tumorigenic effects of IL-6 without affecting its physiological role in infection defence and tissue regeneration.

## References

[1] Hirano T, Yasukawa K, Harada H, Taga T, Watanabe Y, Matsuda T (1986). Complementary DNA for a novel human interleukin (BSF-2) that induces B lymphocytes to produce immunoglobulin. Nature.

[2] Gauldie J, Richards C, Harnish D, Lansdorp P, Baumann H (1987). Interferon beta 2/B-cell stimulatory factor type 2 shares identity with monocyte-derived hepatocyte-stimulating factor and regulates the major acute phase protein response in liver cells. Proc Natl Acad Sci U S A.

[3] Rose-John S, Winthrop K, Calabrese L (2017). The role of IL-6 in host defence against infections: immunobiology and clinical implications. Nat Rev Rheumatol.

[4] Jones SA, Jenkins BJ (2018). Recent insights into targeting the IL-6 cytokine family in inflammatory diseases and cancer. Nat Rev Immunol.

[5] Willis EF, KPA MD, Nguyen QH, Garrido AL, Gillespie ER, Harley SBR (2020). Repopulating microglia promote brain repair in an IL-6-dependent manner. Cell.

[6] Wallenius V, Wallenius K, Ahrén B, Rudling M, Carlsten H, Dickson SL (2002). Interleukin-6-deficient mice develop mature-onset obesity. Nat Med.

[7] Kraakman MJ, Kammoun HL, Allen TL, Deswaerte V, Henstridge DC, Estevez E (2015). Blocking IL-6 trans-signaling prevents high-fat diet-induced adipose tissue macrophage recruitment but does not improve insulin resistance. Cell Metab.

[8] Bazan JF (1990). Haemopoietic receptors and helical cytokines. Immunol Today.

[9] Rose-John S (2018) Interleukin-6 Family Cytokines. Cold Spring Harb Perspect Biol 10. 10.1101/cshperspect.a028415 10.1101/cshperspect.a028415 PMC5793756 28620096

[10] Schaper F, Rose-John S (2015). Interleukin-6: biology, signaling and strategies of blockade. Cytokine Growth Factor Rev.

[11] Baumann H, Gauldie J (1994). The acute phase response. Immunol Today.

[12] Hermanns HM (2015). Oncostatin M and interleukin-31: Cytokines, receptors, signal transduction and physiology. Cytokine Growth Factor Rev.

[13] Taniguchi K, Wu L-W, Grivennikov SI, de Jong PR, Lian I, Yu F-X (2015). A gp130-Src-YAP module links inflammation to epithelial regeneration. Nature.

[14] Fazel Modares N, Polz R, Haghighi F, Lamertz L, Behnke K, Zhuang Y (2019). IL-6 Trans-signaling Controls Liver Regeneration After Partial Hepatectomy. Hepatology.

[15] Tsuchida T, Friedman SL (2017). Mechanisms of hepatic stellate cell activation. Nat Rev Gastroenterol Hepatol.

[16] Piobbico D, Bartoli D, Pieroni S, De Luca A, Castelli M, Romani L (2018). Role of IL-17RA in the proliferative priming of hepatocytes in liver regeneration. Cell Cycle (Georgetown, Tex).

[17] Meng F, Wang K, Aoyama T, Grivennikov SI, Paik Y, Scholten D (2012). Interleukin-17 signaling in inflammatory, Kupffer cells, and hepatic stellate cells exacerbates liver fibrosis in mice. Gastroenterology.

[18] Matthews VB, Knight B, Tirnitz-Parker JEE, Boon J, Olynyk JK, Yeoh GCT (2005). Oncostatin M induces an acute phase response but does not modulate the growth or maturation-status of liver progenitor (oval) cells in culture. Exp Cell Res.

[19] Widjaja AA, Singh BK, Adami E, Viswanathan S, Dong J, D’Agostino GA (2019). Inhibiting interleukin 11 signaling reduces hepatocyte death and liver fibrosis, inflammation, and steatosis in mouse models of nonalcoholic steatohepatitis. Gastroenterology.

[20] Dong J, Viswanathan S, Adami E, Singh BK, Chothani SP, Ng B (2021). Hepatocyte-specific IL11 cis-signaling drives lipotoxicity and underlies the transition from NAFLD to NASH. Nat Commun.

[21] Graf D, Kohlmann C, Haselow K, Gehrmann T, Bode JG, Häussinger D (2006). Bile acids inhibit interleukin-6 signaling via gp130 receptor-dependent and -independent pathways in rat liver. Hepatology.

[22] Müllberg J, Schooltink H, Stoyan T, Günther M, Graeve L, Buse G (1993). The soluble interleukin-6 receptor is generated by shedding. Eur J Immunol.

[23] Matthews V, Schuster B, Schütze S, Bussmeyer I, Ludwig A, Hundhausen C (2003). Cellular cholesterol depletion triggers shedding of the human interleukin-6 receptor by ADAM10 and ADAM17 (TACE). J Biol Chem.

[24] Rose-John S, Heinrich PC (1994). Soluble receptors for cytokines and growth factors: generation and biological function. Biochem J.

[25] Peters M, Blinn G, Solem F, Fischer M (1998). Meyer zum Büschenfelde KH and Rose-John S, In vivo and in vitro activities of the gp130-stimulating designer cytokine Hyper-IL-6. J Immunol.

[26] Liu Z, Sakamoto T, Ezure T, Yokomuro S, Murase N, Michalopoulos G (1998). Interleukin-6, hepatocyte growth factor, and their receptors in biliary epithelial cells during a type I ductular reaction in mice: interactions between the periductal inflammatory and stromal cells and the biliary epithelium. Hepatology.

[27] Xiang D-M, Sun W, Ning B-F, Zhou T-F, Li X-F, Zhong W (2018). The HLF/IL-6/STAT3 feedforward circuit drives hepatic stellate cell activation to promote liver fibrosis. Gut.

[28] Schumacher N, Yan K, Gandraß M, Müller M, Krisp C, Häsler R et al (2021) Cell-autonomous hepatocyte-specific GP130 signalling is sufficient to trigger a robust innate immune response in mice. J Hepatol 74:407–418. 10.1016/j.jhep.2020.09.021 10.1016/j.jhep.2020.09.021 32987028

[29] Schmidt-Arras D, Rose-John S (2016). IL-6 pathway in the liver: from physiopathology to therapy. J Hepatol.

[30] Kopf M, Baumann H, Freer G, Freudenberg M, Lamers M, Kishimoto T (1994). Impaired immune and acute-phase responses in interleukin-6-deficient mice. Nature.

[31] Streetz KL, Tacke F, Leifeld L, Wüstefeld T, Graw A, Klein C (2003). Interleukin 6/gp130-dependent pathways are protective during chronic liver diseases. Hepatology.

[32] Streetz KL, Wüstefeld T, Klein C, Kallen K-J, Tronche F, Betz UAK (2003). Lack of gp130 expression in hepatocytes promotes liver injury. Gastroenterology.

[33] Sander LE, Sackett SD, Dierssen U, Beraza N, Linke RP, Müller M (2010). Hepatic acute-phase proteins control innate immune responses during infection by promoting myeloid-derived suppressor cell function. J Exp Med.

[34] Peters M, Odenthal M, Schirmacher P, Blessing M, Fattori E, Ciliberto G (1997). Soluble IL-6 receptor leads to a paracrine modulation of the IL-6-induced hepatic acute phase response in double transgenic mice. J Immunol (Baltimore, Md: 1950).

[35] Lemmers A, Gustot T, Durnez A, Evrard S, Moreno C, Quertinmont E (2009). An inhibitor of interleukin-6 trans-signalling, sgp130, contributes to impaired acute phase response in human chronic liver disease. Clin Exp Immunol.

[36] Schwerd T, Twigg SRF, Aschenbrenner D, Manrique S, Miller KA, Taylor IB (2017). A biallelic mutation in IL6ST encoding the GP130 co-receptor causes immunodeficiency and craniosynostosis. J Exp Med.

[37] Spencer S, Köstel Bal S, Egner W, Lango Allen H, Raza SI, Ma CA (2019). Loss of the interleukin-6 receptor causes immunodeficiency, atopy, and abnormal inflammatory responses. J Exp Med.

[38] Lee P, Peng H, Gelbart T, Wang L, Beutler E (2005). Regulation of hepcidin transcription by interleukin-1 and interleukin-6. Proc Natl Acad Sci U S A.

[39] Pietrangelo A, Dierssen U, Valli L, Garuti C, Rump A, Corradini E (2007). STAT3 is required for IL-6-gp130-dependent activation of hepcidin in vivo. Gastroenterology.

[40] Klein C, Wüstefeld T, Assmus U, Roskams T, Rose-John S, Müller M (2005). The IL-6-gp130-STAT3 pathway in hepatocytes triggers liver protection in T cell-mediated liver injury. J Clin Invest.

[41] Trautwein C, Rakemann T, Niehof M, Rose-John S, Manns M (1996). Acute-phase response factor, increased binding, and target gene transcription during liver regeneration. Gastroenterology.

[42] Cressman DE, Greenbaum LE, DeAngelis RA, Ciliberto G, Furth EE, Poli V (1996). Liver failure and defective hepatocyte regeneration in interleukin-6-deficient mice. Science.

[43] Zimmers TA, McKillop IH, Pierce RH, Yoo J-Y, Koniaris LG (2003). Massive liver growth in mice induced by systemic interleukin 6 administration. Hepatology.

[44] Gruber S, Straub BK, Ackermann PJ, Wunderlich CM, Mauer J, Seeger JM (2013). Obesity promotes liver carcinogenesis via Mcl-1 stabilization independent of IL-6Rα signaling. Cell Rep.

[45] Bergmann J, Müller M, Baumann N, Reichert M, Heneweer C, Bolik J (2017). IL-6 trans-signaling is essential for the development of hepatocellular carcinoma in mice. Hepatology.

[46] Nechemia-Arbely Y, Shriki A, Denz U, Drucker C, Scheller J, Raub J (2011). Early hepatocyte DNA synthetic response posthepatectomy is modulated by IL-6 trans-signaling and PI3K/AKT activation. J Hepatol.

[47] Chou C-H, Lai S-L, Chen C-N, Lee P-H, Peng F-C, Kuo M-L (2013). IL-6 regulates Mcl-1L expression through the JAK/PI3K/Akt/CREB signaling pathway in hepatocytes: implication of an anti-apoptotic role during liver regeneration. PLoS One.

[48] Schirmacher P, Peters M, Ciliberto G, Blessing M, Lotz J, Meyer zum Büschenfelde KH (1998). Hepatocellular hyperplasia, plasmacytoma formation, and extramedullary hematopoiesis in interleukin (IL)-6/soluble IL-6 receptor double-transgenic mice. Am J Pathol.

[49] Peters M, Blinn G, Jostock T, Schirmacher P, Meyer zum Büschenfelde KH, Galle PR (2000). Combined interleukin 6 and soluble interleukin 6 receptor accelerates murine liver regeneration. Gastroenterology.

[50] Gewiese-Rabsch J, Drucker C, Malchow S, Scheller J, Rose-John S (2010). Role of IL-6 trans-signaling in CCl-induced liver damage. Biochim Biophys Acta.

[51] Dorner AJ, Goldman SJ, Keith JC (1997). Interleukin-11. BioDrugs.

[52] Trepicchio WL, Bozza M, Bouchard P, Dorner AJ (2001). Protective effect of rhIL-11 in a Murine model of acetaminophen-induced hepatotoxicity. Toxicol Pathol.

[53] Nishina T, Komazawa-Sakon S, Yanaka S, Piao X, Zheng D-M, Piao J-H (2012). Interleukin-11 links oxidative stress and compensatory proliferation. Sci Signal.

[54] Zhu M, Lu B, Cao Q, Wu Z, Xu Z, Li W (2015). IL-11 Attenuates liver ischemia/reperfusion injury (IRI) through STAT3 signaling pathway in mice. PLoS One.

[55] Nakamura K, Nonaka H, Saito H, Tanaka M, Miyajima A (2004). Hepatocyte proliferation and tissue remodeling is impaired after liver injury in oncostatin M receptor knockout mice. Hepatology (Baltimore, Md).

[56] Hamada T, Sato A, Hirano T, Yamamoto T, Son G, Onodera M (2007). Oncostatin M gene therapy attenuates liver damage induced by dimethylnitrosamine in rats. Am J Pathol.

[57] Vollmer S, Kappler V, Kaczor J, Flügel D, Rolvering C, Kato N (2009). Hypoxia-inducible factor 1alpha is up-regulated by oncostatin M and participates in oncostatin M signaling. Hepatology (Baltimore, Md).

[58] Matsuda M, Tsurusaki S, Miyata N, Saijou E, Okochi H, Miyajima A (2018). Oncostatin M causes liver fibrosis by regulating cooperation between hepatic stellate cells and macrophages in mice. Hepatology (Baltimore, Md).

[59] Foglia B, Sutti S, Pedicini D, Cannito S, Bocca C, Maggiora M (2019). Oncostatin M, A Profibrogenic mediator overexpressed in non-alcoholic fatty liver disease, stimulates migration of hepatic myofibroblasts. Cells.

[60] Grivennikov SI, Greten FR, Karin M (2010). Immunity, inflammation, and cancer. Cell.

[61] Hou J, Zhang H, Sun B, Karin M (2020). The immunobiology of hepatocellular carcinoma in humans and mice: basic concepts and therapeutic implications. J Hepatol.

[62] Naugler WE, Sakurai T, Kim S, Maeda S, Kim K, Elsharkawy AM (2007). Gender disparity in liver cancer due to sex differences in MyD88-dependent IL-6 production. Science.

[63] Wan S, Zhao E, Kryczek I, Vatan L, Sadovskaya A, Ludema G (2014). Tumor-associated macrophages produce interleukin 6 and signal via STAT3 to promote expansion of human hepatocellular carcinoma stem cells. Gastroenterology.

[64] Lanaya H, Natarajan A, Komposch K, Li L, Amberg N, Chen L (2014). EGFR has a tumour-promoting role in liver macrophages during hepatocellular carcinoma formation. Nat Cell Biol.

[65] Srivatsa S, Paul MC, Cardone C, Holcmann M, Amberg N, Pathria P (2017). EGFR in tumor-associated myeloid cells promotes development of colorectal cancer in mice and associates with outcomes of patients. Gastroenterology.

[66] Buckley AF, Burgart LJ, Sahai V, Kakar S (2008). Epidermal growth factor receptor expression and gene copy number in conventional hepatocellular carcinoma. Am J Clin Pathol.

[67] Feitelson MA, Pan J, Lian Z (2004). Early molecular and genetic determinants of primary liver malignancy. Surg Clin North Am.

[68] Dapito DH, Mencin A, Gwak G-Y, Pradere J-P, Jang M-K, Mederacke I (2012). Promotion of hepatocellular carcinoma by the intestinal microbiota and TLR4. Cancer Cell.

[69] Park EJ, Lee JH, Yu G-Y, He G, Ali SR, Holzer RG (2010). Dietary and genetic obesity promote liver inflammation and tumorigenesis by enhancing IL-6 and TNF expression. Cell.

[70] Guedj A, Volman Y, Geiger-Maor A, Bolik J, Schumacher N, Künzel S (2020). Gut microbiota shape 'inflamm-ageing' cytokines and account for age-dependent decline in DNA damage repair. Gut.

[71] Schwabe RF, Greten TF (2020). Gut microbiome in HCC - Mechanisms, diagnosis and therapy. J Hepatol.

[72] Vaquero J, Campbell JS, Haque J, McMahan RS, Riehle KJ, Bauer RL (2011). Toll-like receptor 4 and myeloid differentiation factor 88 provide mechanistic insights into the cause and effects of interleukin-6 activation in mouse liver regeneration. Hepatology.

[73] Li W, Xiao J, Zhou X, Xu M, Hu C, Xu X (2015). STK4 regulates TLR pathways and protects against chronic inflammation-related hepatocellular carcinoma. J Clin Invest.

[74] Zhou D, Conrad C, Xia F, Park J-S, Payer B, Yin Y (2009). Mst1 and Mst2 maintain hepatocyte quiescence and suppress hepatocellular carcinoma development through inactivation of the Yap1 oncogene. Cancer Cell.

[75] Wei Y, Lao X-M, Xiao X, Wang X-Y, Wu Z-J, Zeng Q-H (2019). Plasma cell polarization to the immunoglobulin G phenotype in hepatocellular carcinomas involves epigenetic alterations and promotes hepatoma progression in mice. Gastroenterology.

[76] Xu B, Broome U, Ericzon B-G, Sumitran-Holgersson S (2002). High frequency of autoantibodies in patients with primary sclerosing cholangitis that bind biliary epithelial cells and induce expression of CD44 and production of interleukin 6. Gut.

[77] Sahai E, Astsaturov I, Cukierman E, DeNardo DG, Egeblad M, Evans RM (2020). A framework for advancing our understanding of cancer-associated fibroblasts. Nat Rev Cancer.

[78] Roehlen N, Crouchet E, Baumert TF (2020). Liver Fibrosis: Mechanistic Concepts and Therapeutic Perspectives. Cells.

[79] Cheteh EH, Sarne V, Ceder S, Bianchi J, Augsten M, Rundqvist H (2020). Interleukin-6 derived from cancer-associated fibroblasts attenuates the p53 response to doxorubicin in prostate cancer cells. Cell Death Dis.

[80] Zhang M, Yang H, Wan L, Wang Z, Wang H, Ge C (2020). Single-cell transcriptomic architecture and intercellular crosstalk of human intrahepatic cholangiocarcinoma. J Hepatol.

[81] Irvine KM, Skoien R, Bokil NJ, Melino M, Thomas GP, Loo D (2014). Senescent human hepatocytes express a unique secretory phenotype and promote macrophage migration. World J Gastroenterol.

[82] Toshima T, Shirabe K, Fukuhara T, Ikegami T, Yoshizumi T, Soejima Y (2014). Suppression of autophagy during liver regeneration impairs energy charge and hepatocyte senescence in mice. Hepatology.

[83] Tabibian JH, O'Hara SP, Splinter PL, Trussoni CE, LaRusso NF (2014). Cholangiocyte senescence by way of N-ras activation is a characteristic of primary sclerosing cholangitis. Hepatology.

[84] Kang T-W, Yevsa T, Woller N, Hoenicke L, Wuestefeld T, Dauch D (2011). Senescence surveillance of pre-malignant hepatocytes limits liver cancer development. Nature.

[85] Zhou J, Liu M, Sun H, Feng Y, Xu L, Chan AWH (2018). Hepatoma-intrinsic CCRK inhibition diminishes myeloid-derived suppressor cell immunosuppression and enhances immune-checkpoint blockade efficacy. Gut.

[86] Bioulac-Sage P, Rebouissou S, Thomas C, Blanc J-F, Saric J, Sa Cunha A (2007). Hepatocellular adenoma subtype classification using molecular markers and immunohistochemistry. Hepatology.

[87] Rebouissou S, Amessou M, Couchy G, Poussin K, Imbeaud S, Pilati C (2009). Frequent in-frame somatic deletions activate gp130 in inflammatory hepatocellular tumours. Nature.

[88] Pilati C, Amessou M, Bihl MP, Balabaud C, Nhieu JTV, Paradis V (2011). Somatic mutations activating STAT3 in human inflammatory hepatocellular adenomas. J Exp Med.

[89] He G, Dhar D, Nakagawa H, Font-Burgada J, Ogata H, Jiang Y (2013). Identification of liver cancer progenitors whose malignant progression depends on autocrine IL-6 signaling. Cell.

[90] J-h Y, Yang F, Wang F, Ma J-Z, Guo Y-J, Tao Q-F (2014). A long noncoding RNA activated by TGF-β promotes the invasion-metastasis cascade in hepatocellular carcinoma. Cancer Cell.

[91] Hatting M, Spannbauer M, Peng J, Al Masaoudi M, Sellge G, Nevzorova YA (2015). Lack of gp130 expression in hepatocytes attenuates tumor progression in the DEN model. Cell Death Dis.

[92] Barash HR, Gross E, Edrei Y, Ella E, Israel A, Cohen I (2010). Accelerated carcinogenesis following liver regeneration is associated with chronic inflammation-induced double-strand DNA breaks. Proc Natl Acad Sci U S A.

[93] Lanton T, Shriki A, Nechemia-Arbely Y, Abramovitch R, Levkovitch O, Adar R (2017). Interleukin 6-dependent genomic instability heralds accelerated carcinogenesis following liver regeneration on a background of chronic hepatitis. Hepatology.

[94] Geiger-Maor A, Guedj A, Even-Ram S, Smith Y, Galun E, Rachmilewitz J (2015). Macrophages regulate the systemic response to DNA damage by a cell nonautonomous mechanism. Cancer Res.

[95] Heim D, Gil-Ibanez I, Herden J, Parplys AC, Borgmann K, Schmidt-Arras D (2016). Constitutive gp130 activation rapidly accelerates the transformation of human hepatocytes via an impaired oxidative stress response. Oncotarget.

[96] Wang D, Zheng X, Fu B, Nian Z, Qian Y, Sun R (2019). Hepatectomy promotes recurrence of liver cancer by enhancing IL-11-STAT3 signaling. EBioMedicine.

[97] Finkin S, Yuan D, Stein I, Taniguchi K, Weber A, Unger K (2015). Ectopic lymphoid structures function as microniches for tumor progenitor cells in hepatocellular carcinoma. Nat Immunol.

[98] Yokomuro S, Tsuji H, Lunz JG, Sakamoto T, Ezure T, Murase N (2000). Growth control of human biliary epithelial cells by interleukin 6, hepatocyte growth factor, transforming growth factor beta1, and activin A: comparison of a cholangiocarcinoma cell line with primary cultures of non-neoplastic biliary epithelial cells. Hepatology.

[99] Stuhlmann-Laeisz C, Lang S, Chalaris A, Krzysztof P, Enge S, Eichler J (2006). Forced dimerization of gp130 leads to constitutive STAT3 activation, cytokine-independent growth, and blockade of differentiation of embryonic stem cells. Mol Biol Cell.

[100] Scherger AK, Al-Maarri M, Maurer HC, Schick M, Maurer S, Öllinger R et al (2019) Activated gp130 signaling selectively targets B cell differentiation to induce mature lymphoma and plasmacytoma. JCI insight:4, e128435. 10.1172/jci.insight.128435 10.1172/jci.insight.128435 PMC6693839 31391340

[101] Pascut D, Pratama MY, Vo NVT, Masadah R, Tiribelli C (2020). The crosstalk between tumor cells and the microenvironment in hepatocellular carcinoma: the role of exosomal microRNAs and their clinical implications. Cancers.

[102] Refolo MG, Messa C, Guerra V, Carr BI, D’Alessandro R (2020). Inflammatory mechanisms of HCC development. Cancers.

[103] Zhang J-P, Yan J, Xu J, Pang X-H, Chen M-S, Li L (2009). Increased intratumoral IL-17-producing cells correlate with poor survival in hepatocellular carcinoma patients. J Hepatol.

[104] Jiang R, Tan Z, Deng L, Chen Y, Xia Y, Gao Y (2011). Interleukin-22 promotes human hepatocellular carcinoma by activation of STAT3. Hepatology.

[105] Kuang D-M, Xiao X, Zhao Q, Chen M-M, Li X-F, Liu R-X (2014). B7-H1-expressing antigen-presenting cells mediate polarization of protumorigenic Th22 subsets. J Clin Invest.

[106] Tan H, Wang S, Zhao L (2017). A tumour-promoting role of Th9 cells in hepatocellular carcinoma through CCL20 and STAT3 pathways. Clin Exp Pharmacol Physiol.

[107] Zhou L, Ivanov II, Spolski R, Min R, Shenderov K, Egawa T (2007). IL-6 programs T(H)-17 cell differentiation by promoting sequential engagement of the IL-21 and IL-23 pathways. Nat Immunol.

[108] Lee J-Y, Hall JA, Kroehling L, Wu L, Najar T, Nguyen HH (2020). Serum amyloid A proteins induce pathogenic Th17 cells and promote inflammatory disease. Cell.

[109] Chan L-C, Li C-W, Xia W, Hsu J-M, Lee H-H, Cha J-H (2019). IL-6/JAK1 pathway drives PD-L1 Y112 phosphorylation to promote cancer immune evasion. J Clin Invest.

[110] Cheng Y, Li H, Deng Y, Tai Y, Zeng K, Zhang Y (2018). Cancer-associated fibroblasts induce PDL1+ neutrophils through the IL6-STAT3 pathway that foster immune suppression in hepatocellular carcinoma. Cell Death Dis.

[111] Lamano JB, Lamano JB, Li YD, DiDomenico JD, Choy W, Veliceasa D (2019). Glioblastoma-derived IL6 induces immunosuppressive peripheral myeloid cell PD-L1 and promotes tumor growth. Clin Cancer Res.

[112] Liu H, Shen J, Lu K (2017). IL-6 and PD-L1 blockade combination inhibits hepatocellular carcinoma cancer development in mouse model. Biochem Biophys Res Commun.

[113] Tsukamoto H, Fujieda K, Miyashita A, Fukushima S, Ikeda T, Kubo Y (2018). Combined blockade of IL6 and PD-1/PD-L1 signaling abrogates mutual regulation of their immunosuppressive effects in the tumor microenvironment. Cancer Res.

[114] Stein S, Henze L, Poch T, Carambia A, Krech T, Preti M (2020). IL-17A/F enable cholangiocytes to restrict T cell-driven experimental cholangitis by upregulating PD-L1 expression. J Hepatol.

[115] Hutchins NA, Chung C-S, Borgerding JN, Ayala CA, Ayala A (2013). Kupffer cells protect liver sinusoidal endothelial cells from Fas-dependent apoptosis in sepsis by down-regulating gp130. Am J Pathol.

[116] Zhuang P-Y, Wang J-D, Tang Z-H, Zhou X-P, Quan Z-W, Liu Y-B (2015). Higher proliferation of peritumoral endothelial cells to IL-6/sIL-6R than tumoral endothelial cells in hepatocellular carcinoma. BMC Cancer.

[117] Toyoshima Y, Kitamura H, Xiang H, Ohno Y, Homma S, Kawamura H (2019). IL6 Modulates the immune status of the tumor microenvironment to facilitate metastatic colonization of colorectal cancer cells. Cancer Immunol Res.

[118] Lee JW, Stone ML, Porrett PM, Thomas SK, Komar CA, Li JH (2019). Hepatocytes direct the formation of a pro-metastatic niche in the liver. Nature.

[119] Yamashita T, Honda M, Nio K, Nakamoto Y, Yamashita T, Takamura H (2010). Oncostatin m renders epithelial cell adhesion molecule-positive liver cancer stem cells sensitive to 5-Fluorouracil by inducing hepatocytic differentiation. Cancer Res.

[120] Peng Z-P, Jiang Z-Z, Guo H-F, Zhou M-M, Huang Y-F, Ning W-R (2020). Glycolytic activation of monocytes regulates the accumulation and function of neutrophils in human hepatocellular carcinoma. J Hepatol.

[121] Song M, He J, Pan Q-Z, Yang J, Zhao J, Zhang Y-J (2021). Cancer-associated fibroblast-mediated cellular crosstalk supports hepatocellular carcinoma progression. Hepatology.

[122] Hu X, Zhao Y, He X, Li J, Wang T, Zhou W (2008). Ciliary neurotrophic factor receptor alpha subunit-modulated multiple downstream signaling pathways in hepatic cancer cell lines and their biological implications. Hepatology (Baltimore, Md).

[123] Luo Q, Zhang Y, Wang N, Jin G, Jin H, Gu D (2015). Leukemia inhibitory factor receptor is a novel immunomarker in distinction of well-differentiated HCC from dysplastic nodules. Oncotarget.

[124] Kao J-T, Feng C-L, Yu C-J, Tsai S-M, Hsu P-N, Chen Y-L (2015). IL-6, through p-STAT3 rather than p-STAT1, activates hepatocarcinogenesis and affects survival of hepatocellular carcinoma patients: a cohort study. BMC Gastroenterol.

[125] Rolvering C, Zimmer AD, Kozar I, Hermanns HM, Letellier E, Vallar L (2017). Crosstalk between different family members: IL27 recapitulates IFNγ responses in HCC cells, but is inhibited by IL6-type cytokines. Biochimica Et Biophysica Acta Molec Cell Res.

[126] Rolvering C, Zimmer AD, Ginolhac A, Margue C, Kirchmeyer M, Servais F (2018). The PD-L1- and IL6-mediated dampening of the IL27/STAT1 anticancer responses are prevented by α-PD-L1 or α-IL6 antibodies. J Leukoc Biol.

[127] Lo C-H, Chang C-M, Tang S-W, Pan W-Y, Fang C-C, Chen Y (2010). Differential antitumor effect of interleukin-12 family cytokines on orthotopic hepatocellular carcinoma. J Gene Med.

[128] Henderson NC, Rieder F, Wynn TA (2020). Fibrosis: from mechanisms to medicines. Nature.

[129] Kisseleva T, Brenner D (2020). Molecular and cellular mechanisms of liver fibrosis and its regression. Nat Rev Gastroenterol Hepatol.

[130] Ezure T, Sakamoto T, Tsuji H, Lunz JG, Murase N, Fung JJ (2000). The development and compensation of biliary cirrhosis in interleukin-6-deficient mice. Am J Pathol.

[131] Miller AM, Wang H, Bertola A, Park O, Horiguchi N, Ki SH (2011). Inflammation-associated interleukin-6/signal transducer and activator of transcription 3 activation ameliorates alcoholic and nonalcoholic fatty liver diseases in interleukin-10-deficient mice. Hepatology.

[132] Hou X, Yin S, Ren R, Liu S, Yong L, Liu Y et al (2020) Myeloid cell-specific IL-6 signaling promotes miR-223-enriched exosome production to attenuate NAFLD-associated fibrosis. Hepatology. 10.1002/hep.31658 10.1002/hep.31658 PMC8141545 33236445

[133] Deng Y-R, Ma H-D, Tsuneyama K, Yang W, Wang Y-H, Lu F-T (2013). STAT3-mediated attenuation of CCl4-induced mouse liver fibrosis by the protein kinase inhibitor sorafenib. J Autoimmun.

[134] Shigekawa M, Takehara T, Kodama T, Hikita H, Shimizu S, Li W (2011). Involvement of STAT3-regulated hepatic soluble factors in attenuation of stellate cell activity and liver fibrogenesis in mice. Biochem Biophys Res Commun.

[135] Gajalakshmi P, Majumder S, Viebahn CS, Swaminathan A, Yeoh GC, Chatterjee S (2017). Interleukin-6 secreted by bipotential murine oval liver stem cells induces apoptosis of activated hepatic stellate cells by activating NF-κB-inducible nitric oxide synthase signaling. Biochem Cell Biol.

[136] Stärkel P, Schnabl B, Leclercq S, Komuta M, Bataller R, Argemi J (2019). Deficient IL-6/Stat3 signaling, High TLR7, and type I interferons in early human alcoholic liver disease: a triad for liver damage and fibrosis. Hepatol Commun.

[137] Li L, Duan C, Zhao Y, Zhang X, Yin H, Wang T (2017). Preventive effects of interleukin-6 in lipopolysaccharide/d-galactosamine induced acute liver injury via regulating inflammatory response in hepatic macrophages. Int Immunopharmacol.

[138] Jepsen P, Kraglund F, West J, Villadsen GE, Sørensen HT, Vilstrup H (2020). Risk of hepatocellular carcinoma in Danish outpatients with alcohol-related cirrhosis. J Hepatol.

[139] Hong F, Kim W-H, Tian Z, Jaruga B, Ishac E, Shen X (2002). Elevated interleukin-6 during ethanol consumption acts as a potential endogenous protective cytokine against ethanol-induced apoptosis in the liver: involvement of induction of Bcl-2 and Bcl-x(L) proteins. Oncogene.

[140] El-Assal O, Hong F, Kim W-H, Radaeva S, Gao B (2004). IL-6-deficient mice are susceptible to ethanol-induced hepatic steatosis: IL-6 protects against ethanol-induced oxidative stress and mitochondrial permeability transition in the liver. Cell Mol Immunol.

[141] Chiang K-C, Chang K-S, Hsu S-Y, Sung H-C, Feng T-H, Chao M (2020). Human heme oxygenase-1 induced by Interleukin-6 via JAK/STAT3 pathways is a tumor suppressor gene in hepatoma cells. Antioxidants.

[142] Shih P-C (2020). Revisiting the development of small molecular inhibitors that directly target the signal transducer and activator of transcription 3 (STAT3) domains. Life Sci.

[143] Kallunki T, Olsen OD, Jäättelä M (2013). Cancer-associated lysosomal changes: friends or foes?. Oncogene.

[144] Sargeant TJ, Lloyd-Lewis B, Resemann HK, Ramos-Montoya A, Skepper J, Watson CJ (2014). Stat3 controls cell death during mammary gland involution by regulating uptake of milk fat globules and lysosomal membrane permeabilization. Nat Cell Biol.

[145] Lee TKW, Castilho A, Cheung VCH, Tang KH, Ma S, Ng IOL (2011). CD24(+) liver tumor-initiating cells drive self-renewal and tumor initiation through STAT3-mediated NANOG regulation. Cell Stem Cell.

[146] D'Amico S, Shi J, Martin BL, Crawford HC, Petrenko O, Reich NC (2018). STAT3 is a master regulator of epithelial identity and KRAS-driven tumorigenesis. Genes Dev.

[147] Couto JP, Daly L, Almeida A, Knauf JA, Fagin JA, Sobrinho-Simões M (2012). STAT3 negatively regulates thyroid tumorigenesis. Proc Natl Acad Sci U S A.

[148] de la Iglesia N, Konopka G, Puram SV, Chan JA, Bachoo RM, You MJ (2008). Identification of a PTEN-regulated STAT3 brain tumor suppressor pathway. Genes Dev.

[149] Musteanu M, Blaas L, Mair M, Schlederer M, Bilban M, Tauber S (2010). Stat3 is a negative regulator of intestinal tumor progression in Apc(Min) mice. Gastroenterology.

[150] Lee J, Kim JCK, Lee S-E, Quinley C, Kim H, Herdman S (2012). Signal transducer and activator of transcription 3 (STAT3) protein suppresses adenoma-to-carcinoma transition in Apcmin/+ mice via regulation of Snail-1 (SNAI) protein stability. J Biol Chem.

[151] Grabner B, Schramek D, Mueller KM, Moll HP, Svinka J, Hoffmann T (2015). Disruption of STAT3 signalling promotes KRAS-induced lung tumorigenesis. Nat Commun.

[152] Pencik J, Schlederer M, Gruber W, Unger C, Walker SM, Chalaris A (2015). STAT3 regulated ARF expression suppresses prostate cancer metastasis. Nat Commun.

[153] Fizazi K, De Bono JS, Flechon A, Heidenreich A, Voog E, Davis NB (2012). Randomised phase II study of siltuximab (CNTO 328), an anti-IL-6 monoclonal antibody, in combination with mitoxantrone/prednisone versus mitoxantrone/prednisone alone in metastatic castration-resistant prostate cancer. Eur J Cancer.

[154] Jhan J-R, Andrechek ER (2016). Stat3 accelerates Myc induced tumor formation while reducing growth rate in a mouse model of breast cancer. Oncotarget.

[155] Jia X, Lu S, Zeng Z, Liu Q, Dong Z, Chen Y (2020). Characterization of gut microbiota, bile acid metabolism, and cytokines in intrahepatic cholangiocarcinoma. Hepatology.

[156] Lv B, Ma L, Tang W, Huang P, Yang B, Wang L (2018). FXR acts as a metastasis suppressor in intrahepatic cholangiocarcinoma by inhibiting IL-6-induced epithelial-mesenchymal transition. Cell Physiol Biochem.

[157] Kleinegger F, Hofer E, Wodlej C, Golob-Schwarzl N, Birkl-Toeglhofer AM, Stallinger A (2019). Pharmacologic IL-6Rα inhibition in cholangiocarcinoma promotes cancer cell growth and survival. Biochimica Et Biophysica Acta.

[158] Gorgoulis V, Adams PD, Alimonti A, Bennett DC, Bischof O, Bishop C (2019). Cellular senescence: defining a path forward. Cell.

[159] Basisty N, Kale A, Jeon OH, Kuehnemann C, Payne T, Rao C (2020). A proteomic atlas of senescence-associated secretomes for aging biomarker development. PLoS Biol.

[160] Wang L, Leite de Oliveira R, Wang C, Fernandes Neto JM, Mainardi S, Evers B (2017). High-throughput functional genetic and compound screens identify targets for senescence induction in cancer. Cell Rep.

[161] Wang C, Vegna S, Jin H, Benedict B, Lieftink C, Ramirez C (2019). Inducing and exploiting vulnerabilities for the treatment of liver cancer. Nature.

[162] Sapochnik M, Haedo MR, Fuertes M, Ajler P, Carrizo G, Cervio A (2017). Autocrine IL-6 mediates pituitary tumor senescence. Oncotarget.

[163] García-Sáinz JA (1988). 'Inhibitory' receptors and ion channel effectors. Trends Pharmacol Sci.

[164] Wolter K, Zender L (2020). Therapy-induced senescence - an induced synthetic lethality in liver cancer?. Nat Rev Gastroenterol Hepatol.

[165] Llovet JM, Ricci S, Mazzaferro V, Hilgard P, Gane E, Blanc J-F (2008). Sorafenib in advanced hepatocellular carcinoma. N Engl J Med.

[166] Kudo M, Finn RS, Qin S, Han K-H, Ikeda K, Piscaglia F (2018). Lenvatinib versus sorafenib in first-line treatment of patients with unresectable hepatocellular carcinoma: a randomised phase 3 non-inferiority trial. Lancet.

[167] Finn RS, Qin S, Ikeda M, Galle PR, Ducreux M, Kim T-Y (2020). Atezolizumab plus bevacizumab in unresectable hepatocellular carcinoma. N Engl J Med.

[168] Ioannou GN (2020). HCC surveillance after SVR in patients with F3/F4 fibrosis. J Hepatol.

[169] Forner A, Reig M, Bruix J (2018). Hepatocellular carcinoma. Lancet.

[170] Katzenellenbogen M, Mizrahi L, Pappo O, Klopstock N, Olam D, Jacob-Hirsch J (2007). Molecular mechanisms of liver carcinogenesis in the mdr2-knockout mice. Mol Cancer Res.

[171] Ahmed M, Brace CL, Lee FT, Goldberg SN (2011). Principles of and advances in percutaneous ablation. Radiology.

[172] Ganne-Carrié N, Nault J-C, Ziol M, N'Kontchou G, Nahon P, Grando V (2014). Predicting recurrence following radiofrequency percutaneous ablation for hepatocellular carcinoma. Hepatic Oncol.

[173] Ahmed M, Kumar G, Navarro G, Wang Y, Gourevitch S, Moussa MH (2015). Systemic siRNA nanoparticle-based drugs combined with radiofrequency ablation for cancer therapy. PLoS One.

[174] Rozenblum N, Zeira E, Bulvik B, Gourevitch S, Yotvat H, Galun E (2015). Radiofrequency ablation: inflammatory changes in the periablative zone can induce global organ effects, including liver regeneration. Radiology.

[175] Rozenblum N, Zeira E, Scaiewicz V, Bulvik B, Gourevitch S, Yotvat H (2015). Oncogenesis: an "off-target" effect of radiofrequency ablation. Radiology.

[176] Ahmed M, Kumar G, Moussa M, Wang Y, Rozenblum N, Galun E (2016). Hepatic radiofrequency ablation-induced stimulation of distant tumor growth is suppressed by c-Met inhibition. Radiology.

[177] Bulvik BE, Rozenblum N, Gourevich S, Ahmed M, Andriyanov AV, Galun E (2016). Irreversible electroporation versus radiofrequency ablation: a comparison of local and systemic effects in a small-animal model. Radiology.

[178] Kumar G, Goldberg SN, Wang Y, Velez E, Gourevitch S, Galun E (2017). Hepatic radiofrequency ablation: markedly reduced systemic effects by modulating periablational inflammation via cyclooxygenase-2 inhibition. Eur Radiol.

[179] Ahmed M, Kumar G, Gourevitch S, Levchenko T, Galun E, Torchilin V (2018). Radiofrequency ablation (RFA)-induced systemic tumor growth can be reduced by suppression of resultant heat shock proteins. Int J Hyperth.

[180] Kumar G, Goldberg SN, Gourevitch S, Levchenko T, Torchilin V, Galun E (2018). Targeting STAT3 to suppress systemic pro-oncogenic effects from hepatic radiofrequency ablation. Radiology.

[181] Liao H, Ahmed M, Markezana A, Zeng G, Stechele M, Galun E (2020). Thermal ablation induces transitory metastatic growth by means of the STAT3/c-Met molecular pathway in an intrahepatic colorectal cancer mouse model. Radiology.

[182] Markezana A, Ahmed M, Kumar G, Zorde-Khvalevsky E, Rozenblum N, Galun E (2020). Moderate hyperthermic heating encountered during thermal ablation increases tumor cell activity. Int J Hyperth.

